# Pharmacokinetic and pharmacodynamic considerations for antifungal therapy optimisation in the treatment of intra-abdominal candidiasis

**DOI:** 10.1186/s13054-023-04742-w

**Published:** 2023-11-20

**Authors:** Emmanuel Novy, Claire Roger, Jason A. Roberts, Menino Osbert Cotta

**Affiliations:** 1 University of Queensland Centre for Clinical Research (UQCCR), Faculty of Medicine, The University of Queensland, Royal Brisbane & Women’s Hospital Campus Herston, Brisbane, QLD 4029 Australia; 2 Department of Anaesthesiology, Critical Care and Peri-Operative Medicine, University Hospital of Nancy, Nancy, France; 3 Université de Lorraine, SIMPA, 54500 Nancy, France; 4 Department of Anesthesiology, Critical Care, Pain and Emergency Medicine, Nimes University Hospital, Place du Professeur Robert Debré, 30029 Nîmes Cedex 9, France; 5 UR UM103 IMAGINE, Univ Montpellier, Montpellier, France; 6 Department of Intensive Care Medicine and Pharmacy, Royal Brisbane and Women’s Hospital, Brisbane, QLD Australia; 7 Herston Infectious Diseases Institute (HeIDI), Metro North Health, Brisbane, Australia

**Keywords:** Intra-abdominal candidiasis, Antifungal, Pharmacokinetic, Pharmacodynamic, Critically ill patients

## Abstract

Intra-abdominal candidiasis (IAC) is one of the most common of invasive candidiasis observed in critically ill patients. It is associated with high mortality, with up to 50% of deaths attributable to delays in source control and/or the introduction of antifungal therapy. Currently, there is no comprehensive guidance on optimising antifungal dosing in the treatment of IAC among the critically ill. However, this form of abdominal sepsis presents specific pharmacokinetic (PK) alterations and pharmacodynamic (PD) challenges that risk suboptimal antifungal exposure at the site of infection in critically ill patients. This review aims to describe the peculiarities of IAC from both PK and PD perspectives, advocating an individualized approach to antifungal dosing. Additionally, all current PK/PD studies relating to IAC are reviewed in terms of strength and limitations, so that core elements for the basis of future research can be provided.

**Supplementary Information:**

The online version contains supplementary material available at 10.1186/s13054-023-04742-w.

## Highlights

- Intra-abdominal candidiasis presents specific pharmacokinetic (PK) and pharmacodynamic (PD) challenges where suboptimal antifungal concentrations are likely to occur leading to high risk of treatment failure.
- The intra-abdominal cavity has been highlighted as a hidden reservoir for resistance to antifungals including echinocandins.
- To date, all antifungal PK/PD studies in intra-abdominal candidiasis have enrolled small cohorts and have only provided post-operative antifungal concentrations analysis.
- Based on current evidence, high dosing regimens of antifungals should be strongly considered, especially at the onset of infection.
- The place of new antifungals (rezafungin, ibrexafungerp) requires more robust clinical studies including PK/PD analysis in critically ill patients.

**Supplementary Information:**

The online version contains supplementary material available at 10.1186/s13054-023-04742-w.

## Introduction

Intra-abdominal candidiasis (IAC) is defined as the isolation of *Candida* from a sterile intra-abdominal sample along with symptoms of intra-abdominal infection [1, 2]. Current epidemiological data quantifies IAC having a cumulative incidence of 1.84 cases per 1,000 ICU admissions and a mortality up to 50% [3, 4]. *C. albicans* accounts for most infections followed by non-albicans species such as *C. glabrata* and *C. parapsilosis* [5, 6].

The management of IAC requires initiating early and adequate source control, and prompt initiation of antifungal therapy [7, 8]. Current guidelines recommend echinocandins as the first-line agent for empirical treatment of IAC in critically ill patients [8–10]. Fluconazole has been recommended for clinically stable patients with no recent exposure to azoles in the setting of fluconazole-susceptible pathogens [10, 11]. Second-line agents include the liposomal formulation of amphotericin B [10, 11] and voriconazole [12]. However, these guidelines do not provide any specific recommendations regarding alternative dosing regimens in the critically ill [7]. Indeed, only standardized antifungal dosing regimens are proposed. Of note, specific guidelines in cases of critically ill obese patients or those receiving extracorporeal support are lacking, even though IAC is common to these situations which are associated with difficult-to-predict antifungal concentrations [13]. The considerable pharmacokinetics (PK) inter-variability of antifungals was highlighted in the multinational Defining Antibiotic Levels in Intensive care Unit (DALI) study [14].

Most of the studies providing antifungal dosing recommendations for invasive candidiasis generally include more patients with candidemia than IAC [15]. Thus, most studies describe antifungal exposure in the central compartment (i.e., blood). However, it has been demonstrated that many antifungals diffuse poorly into intra-abdominal collections [13, 16]. Therefore, considering that critically ill patients may have lower plasma antifungal concentrations compared to other population groups, the risk of suboptimal concentrations at the site of infection may be even higher [17, 18].

This review explores the pharmacokinetic and pharmacodynamic considerations for antifungal therapy in the treatment of IAC among critically ill patients. Additionally, it proposes dose optimisation strategies of the most used antifungals in the treatment of IAC.

## Pharmacokinetic considerations

Critically ill patients have severe pathophysiological changes driven by a change in the volume of distribution (Vd) and/or modified renal and/or hepatic clearances [19]. Altered Vd is characterized by the expansion of the interstitial space secondary to an increase in capillary permeability, vascular filling, and change in protein binding. “Capillary leakage” is caused by systemic inflammation, a common feature during sepsis that is even more pronounced in septic shock. In critically ill patients with intra-abdominal infections, sepsis and septic shock affect more than 60% and 30% of them, respectively [20]. Abdominal sepsis is also associated with a cytokine “storm”, further aggravating the patient’s inflammatory state compared to sepsis of other origins [21]. Besides, abdominal surgery induced-trauma results in post-operative inflammation [22, 23]. The degree of post-operative inflammation depends on the surgical approach [24]. Laparotomy, which is the most commonly used [25], is more aggressive and thereby highly “inflammatory” compared to laparoscopic surgery. In addition to this “high” inflammatory state, abdominal sepsis is characterized by severe hypovolemia caused by a high prevalence of fever and other sources of fluid loss including anorexia, nausea, and vomiting [26]. Surgery also provides changes in extracellular fluid space mostly driven by neuroendocrine mechanisms. Indeed, part of the endocrine response to surgery involves the increased secretion of antidiuretic hormone and aldosterone leading to salt and water retention [27]. Sequestration of fluids may also occur due to large volume of fluids lost into a distended gut or the peritoneal cavity [28]. Thus, vascular filling is potentially massive in the sickest of these patients, with up to 10 L administered during the first days of fluid resuscitation [17, 29]. An increase in the drug Vd equates to insufficient drug concentrations within the central compartment (i.e. plasma). Lastly, surgery itself affects drug distribution by changes in blood volume (blood and fluid losses during laparotomy) and altered regional perfusion secondary to an increase in circulating catecholamines induced by surgical trauma [28]. Local inflammation combined with decreases in regional blood flow contribute to drug diffusion impairment.

During the post-operative period, interventions that utilise extracorporeal circuits such as renal replacement therapy (RRT) or extracorporeal membrane oxygenation (ECMO) may also contribute to changes in drug clearance depending on the physicochemical properties of the drug [30, 31]. In addition, alterations of protein binding are highly frequent in surgical ICU patients because of a physiological decrease of pre-albumin and albumin in response to an increase of inflammatory protein. Hypoalbuminemia is worsened by vascular filling, malnutrition and a catabolic state, frequent situations seen in post-operative surgical patients [32]. Lastly, indwelling abdominal drains left in situ post-operatively also contribute to an increased elimination of drug [33]. Indeed, previous studies have reported that the increase in drug clearance is proportional to drainage output [34]. Therefore, critically ill patients with IAC have significant drug PK alteration that potentially predisposes them to inadequate drug concentrations that can be exacerbated by surgical interventions during their ICU admission.

## Pharmacokinetic properties of parenteral antifungals used in intra-abdominal candidiasis

Table 1 summarises the PK of antifungal agents used in the treatment of intra-abdominal candidiasis. Dosing regimens and therapeutic ranges for therapeutic drug monitoring (TDM) are proposed based on current evidence.

**Table 1:** Overview of pharmacokinetic parameters and dosing regimens of antifungals for intra-abdominal candidiasis

	First-line agents				Second-line agents	
	Fluconazole	Caspofungin	Anidulafungin	Micafungin	Liposomal amphotericin B	Voriconazole
*Healthy volunteer*						
Recommended dosing regimen	LD 800 mg (12 mg/kg) MD 400 mg (6 mg/kg)	LD 70 mg MD 50 mg (70 if BW > 80 kg)	LD 200 mg MD 100 mg	100 mg	3–5 mg/kg	LD 6 mg/kg q12h MD 4 mg/kg q12h
C_max_ (mg/L)	9	9.5–12	7–8	8–18	58–90	3.1–4.7
AUC (mg/h/L)	90–100	98	110	66	713	13–33
Vd (L/kg)	0.7–0.8	0.15–2	0.6	0.2	0.2–1.6	4.6
Protein binding (%)	12	92.4–96.5	99.0	99.9	95–99	58
Half-life (h)	25–40	9–11	40–50	11–20	13–34	6
CL (mL/min)	15–46	10–12	1	12.8–14	11	250
*Critically ill patient*						
Proposed dosing regimen	LD 12 mg/kg MD 6 mg/kg	LD 100–140 mg MD 50–70 mg	LD 200 mg MD 100 mg	LD 150 mg MD 150 mg	5 mg/kg	LD 6 mg/kg q12h MD 4 mg/kg q12h
Vd (L/kg)	1.2–1.4	0.1	0.5	3.45	0.42	4.3
Half-life (h)	30–75	18.4	31.2	14.8	13	13–21
CL (mL/min)	13–36	13	17.1	12.2	0.36–1.4	61–99
Renal impairment	Decreased dose by 50% when renal CL < 50 mL/min	No dose adjustment			No dose adjustment	Switch to oral formulation when creatinine CL < 50 ml/min
CRRT	LD 12 mg/kg MD 6 mg/kg Consider increasing the dose if ultrafiltration rate > 2L/h Or 300–400 mg q12	No dose adjustment			No dose adjustment	
Hepatic impairment	Limited date, no specific recommendations	Child Pugh 7–9, decrease MD to 35 mg q24h	No dose adjustment	No dose adjustment	Limited data, no specific recommendations	Reduced maintenance dose of 50%
Hypoalbuminemia	No data	MD 50–70 mg according to the MIC	Reduced AUC but not defined dose	Reduced AUC but not defined dose	No data	Considered unbound concentration to be 50% higher than the total measured concentration
Obesity	Avoid fixed–dose, use weight-based dosing (TBW) LD 12 mg/kg MD 6–12 mg/kg	Increased the LD and MD by at least 25 to 50% when body weight > 80–120 kg LD 2 mg/kg MD 1.25 mg/kg	Increased the LD and MD by 25% in patients > 140 kg	Increased the MD from 100 to 300 mg according to the MIC when body weight > 125 kg	Avoid fixed–dose, use weight-based dosing (TBW) 5 mg/kg	Avoid fixed–dose, use weight-based dosing (ABW) (based on non-ICU patients)
ECMO	No data in adult patients	Increased the MD to 70 mg	No dose adjustment	Increased the MD to 150 mg	Only case reports suggesting increasing of the MD and/or change for extended infusion or switch for amphotericin B deoxycholate	Circuit loss reported but no specific recommendations
*Therapeutic drug monitoring*						
PK/PD target	*f*AUC_0-24_/MIC ≥ 100	AUC_0-24_/MIC > 250 (*C. tropicalis*/*krusei*) 450 (*C.glabrata*)–865 (*C.albicans*)–1185 (*C.parapsilosis*)	AUC_24_/MIC > 2000 (*C.parapsilosis*)–3000 (*C.glabrata*)–9000 (*C.albicans*)	AUC_24_/MIC > 3000 (*C.glabrata/parapsilosis*)–10,000 (*C.albicans*)	Not defined	*f*AUC_24_/MIC > 25–100
Therapeutic range (AUC) mg/h/L	> 400	> 100	110	> 130	Not defined	Not defined
Therapeutic range (C_min_) mg/L	10–15	1–10	1–10	1–10	Not correlated with efficacy	2–6 (MIC of 1 mg/L)

According to [13, 31, 35–46, 52–69, 77–87, 89–98]

ABW: adjusted body weight; AUC: area under the curve; CL: clearance; C_min_: minimal concentration; C_max_: maximal concentration; CRRT: continuous renal replacement therapy; ECMO: extracorporeal membrane oxygenation; *f*AUC: AUC of the free concentration; LD: loading dose; MD: maintain dose; MIC: minimal inhibitory concentration; PK/PD: pharmacokinetic and pharmacodynamic; q12: every 12 h; TBW: total body weight; Vd: volume of distribution

### First-line agents

#### Fluconazole

Fluconazole is a triazole which inhibits the 14-α-demethylase which is an enzyme required for conversion of lanosterol into ergosterol. It has low plasma protein binding of 12% and is a weakly basic, highly polar, hydrophilic (LogP 0.5) drug with a small molecular weight [35]. These physicochemical properties expose fluconazole to unpredictable concentrations in the critically ill patient [36, 37]. Indeed, hydrophilic, low protein-binding and low molecular weight are highly influenced by increased Vd and renal clearance; leading to low drug exposure [19].

Fluconazole exhibits concentration- and time-dependent antifungal activity with a prolonged post-antifungal effect. The predictive PK/PD index associated with maximal fungal killing is the ratio of free-drug area under the concentration time curve (from 0 to 24 h) to minimum inhibitory concentration of the fungal organism (*f*AUC_0-24_/MIC). An *f*AUC_0-24_/MIC of greater than 100 is associated with optimal fungicidal activity and positive outcomes in critically ill patients [38, 39]. Current dosing regimens in critically ill patients with normal renal function recommend a loading dose of 12 mg/kg intravenously followed by a maintenance dose of 6—12 mg/kg/day [40]. Maintenance doses of up to 18 mg/kg per day have been proposed [41]. Factors associated with suboptimal fluconazole exposure include obesity, high renal clearance and patients undergoing continuous RRT [42, 43]. In obese patients, total body weight has been used to calculate doses [44]. In the setting of RRT, adjusting the maintenance dose to 800 mg (400 mg q12h) has been suggested [45]. In contrast, ECMO by itself does not seem to influence fluconazole PK [31]. Indeed, PK alteration under ECMO is mostly related to lipophilicity or changes in Vd and to date, no sequestration in the ECMO circuit has been demonstrated with fluconazole. However, data in adult patients are scarce and more studies are warranted to better address optimal dosing of fluconazole in patients undergoing ECMO.

Regarding abdominal diffusion of fluconazole, very few data are available [46]. Sinnollareddy et al. [47] have measured subcutaneous concentrations of fluconazole in critically ill patients and reported an AUC_0-24_ 50% lower than in plasma. A mini-case series of three liver transplant patients reported that the ascites-to-plasma ratio of fluconazole was 0.85 [48]. A case report of a patient with cholecystitis showed that biliary fluconazole concentrations were 50% lower than in the plasma [49]. Lastly, relatively good penetration (88 to 91%) of fluconazole in the pancreas has been reported [50]. This is of significance given that pancreatic necrosis is a frequent source of IAC. Therefore, if tissue penetration based on small studies advocate for relatively good penetration of fluconazole (> 50%), physicochemical properties of fluconazole expose it to suboptimal plasma concentrations in critically ill patients. As low plasma concentrations are associated with even lower tissue concentrations for hydrophilic drugs [51], the risk of suboptimal fluconazole concentrations at the site of infection must be considered very high. Thus, higher doses and TDM should be considered when treating IAC with fluconazole. However, data regarding the therapeutic range of fluconazole are lacking [40, 52, 53]. For instance, a mean trough concentration of 14 mg/L ± 11 mg/L was reported in the DALI study [14]. A trough concentration of 10 to 15 mg/L has been proposed as a basis for fluconazole TDM [52].

#### Echinocandins

##### General considerations

Echinocandins are cyclic hexa-lipopeptides targeting the fungal wall by inhibiting 1.3 beta-d-glucan synthesis [54]. They are fungicidal against most pathogenic species of *Candida* [55].

Echinocandins are highly protein-bound (≥ 90%), hydrophilic molecules which are eliminated through ubiquitous spontaneous degradation. The PK/PD index most frequently reported for echinocandins is AUC/MIC [56, 57]. The AUC/MIC ratios ranged from > 250 (caspofungin/*C. tropicalis*) to > 10,000 (micafungin/*C. albicans*) [58] (Table 1). Thanks to low drug-drug interactions, non-renal elimination, and less extensive hepatic clearance, echinocandins are often considered easy-to-dose drugs [13]. Reported adverse events to date have not established any exposure-related toxicity [56]. Additionally, high doses in the treatment of endocarditis are well tolerated [59]. However, a growing body of evidence has challenged the concept of fixed standard doses [18, 56, 60]. Higher body weights may require higher dosing [61, 62], whilst patients with hypoalbuminemia may have an increased Vd and clearance [13]. Most studies point towards a 20% lower exposures in critically ill patients when compared with healthy volunteers [63–65].

##### Caspofungin

It has been suggested to increase the loading dose to 140 mg in critically ill patients [66]. Likewise, a higher, weight-based dose of 2 mg/kg as a loading dose and 1.25 mg/kg as a maintenance dose have been recommended in critically ill obese patients [67]. Regarding ECMO, caspofungin loss via sequestering within the ECMO circuit was reported, suggesting to increase the loading and 24-hourly maintenance dose to 70 mg, respectively [31, 68].

##### Micafungin

It has been proposed to increase the maintenance dose from 150 mg to between 200 to 300 mg 24-hourly in critically ill obese patients (> 125 kg), depending on the MIC [61]. In patients with ECMO, it has been suggested to increase the micafungin loading and 24-hourly maintenance dose to 150 and 70 mg, respectively [31].

##### Anidulafungin

If suboptimal exposures have been reported (~ 30% lower concentrations compared to healthy volunteers [69]), current data does not define what higher doses should be used [68]. No dosing adjustments are currently recommended for anidulafungin during ECMO.

##### Peritoneal diffusion of echinocandins

Regarding peritoneal diffusion of echinocandins, eight PK studies [57, 70–76] (Table 2) were performed and reported an overall penetration ratio of ~ 30%, knowing that no PK/PD target in the peritoneum has been defined to date. One study also reported highly variable ascites-to-plasma echinocandin penetration ratios ranging from 0.02 to 0.46 in ascitic fluids [48].

**Table 2:** Overview of clinical PK/PD studies reporting abdominal (ascitic and peritoneal fluid) concentrations of antifungals

Author	Type of study	Population	ATF	Samples	Results	Notes
Lin [99]	Prospective monocentric PK study	Adults with suspected or confirmed invasive candidiasis *N* = 19 (4 Surgical)	Voriconazole Prophylaxis (*n* = 9) Treatment (*n* = 10)	Day 1 and then TDM data Day 1: h1, h2, h4, h6, h8, and h12 Peritoneal samples obtained from drain	Low and lower fluctuations of voriconazole concentrations in the PF than in the plasma Penetration ratio: 0.54 (single dose) and 0.67 (multiple doses) 81% of steady state concentration reached the PK/PD target	No IAC Heterogeneity of the population and voriconazole indication Intensive sampling only at Day 1 Peritoneal samples obtained from drain
Tortora [87]	Observational retrospective study	Children with liver transplantation *N* = 6 (5 months–242 months)	Liposomal amphotericin B 3 mg/kg	Plasma: TDM Day 1 to 4 PF Day 1 to 4 Peritoneal samples obtained from drain	Peritoneal concentrations were lower than plasma with a correlation coefficient of 0.72 None of the patient reached the PK/PD target attainment in the PF	No IAC TDM data Peritoneal samples obtained from drain
Garbez [57]	Prospective monocentric PK study Blood (*n* = 159) and peritoneal (*n* = 29) samples	Adults with secondary peritonitis *N* = 11 SAPS II 38 [24–77] SOFA 7 [0–12]	Caspofungin 70 mg then 50 or 70 mg (< > 80 kg)	Day 1, between 3 and 4 PF day 1 h1, h1.5, h2, h4, h6, h12 and h24 Peritoneal samples obtained from drain	High PK variability Penetration ratio: 0.33 Adequate PTA for most susceptible species in patients with Free Fat Mass < 50 kg	IAC = 10% (*n* = 1/10) No unbound concentration Peritoneal samples obtained from drain at day 1 No ATF concentrations during surgery
Welte [74]	Prospective monocentric PK study Blood, ascitic fluid and peritoneal fluid samples	Adults with proven or suspected invasive fungal infections *N* = 29 ANF = 11 CSF = 6 MCF = 13	Caspofungin 70 mg then 50 mg Anidulafungin 200 mg then 100 mg Micafungin 100 mg q24h	Day 1: h4, h8, h12, h18, and h24 Paracentesis on-demand Ascitic fluid from drain	Echinocandin concentrations in ascites fluid were lower than the simultaneous plasma levels	Nine patients with peritonitis with only 2 IAC Highly heterogenous population No unbound concentration Ascitic fluid from drain No ATF concentrations during surgery
Garbez [72]	Prospective monocentric PK study Blood (*n* = 171) and peritoneal (*n* = 42) samples	Adults with secondary peritonitis *N* = 12 SAPS II 40 [29–67] SOFA 5 [1–9]	Micafungin 100 mg q24h	Day 1, between 3 and 5 h1, h1.5, h2, h4, h6, h12 and h24 Peritoneal samples obtained from drain	High PK variability Penetration ratio: 0.25 (Day 1) and 0.4 (Day 3–5) Adequate PTA for most susceptible species in patients with Free Fat Mass < 65 kg	IAC = 50% (*n* = 5/10) No unbound concentration Peritoneal samples obtained from drain No ATF concentrations during surgery
Gioia [73]	Prospective monocentric PK study Blood and peritoneal samples	Adults with PPO *N* = 23 ANF = 11 CSF = 8 MCF = 4	Caspofungin 70 mg then 50 mg Anidulafungin 200 mg then 100 mg Micafungin 100 mg q24h	Day 4 h1, h6, h12, h24h	Most PF ATF concentrations < 1 μg/mL Penetration ratio: 0.3	IAC = 74% (*n* = 17/23) No unbound concentration Peritoneal samples obtained from drain No ATF concentrations during surgery
Pérez Civantos [75]	Prospective multicentric PK study Blood and peritoneal samples	Adults with secondary and tertiary peritonitis *N* = 31 Apache II 22.7 ± 5.9 SOFA 10.3 ± 3.5	Anidulafungin 200 mg then 100 mg q24	Day 2, after LD and 1 MD h1, h3, h6, h12, h18 and h24 Peritoneal samples obtained from drain	ANF exposure PF < plasma Penetration ratio: 0.3	IAC = 12% (*n* = 4/31) No unbound concentration Peritoneal samples obtained from drain No ATF concentrations during surgery
Dupont [71]	Prospective multicentric PK study Blood samples	Adults with complicated IAIs *N* = 14 SAPS II 54 [45–67] SOFA 8 [7–12]	Anidulafungin 200 mg then 100 mg q24	Day 1: T0, Tmax, T24; day 3: T0, Tmax, T24; day 5: T0, Tmax, T3, T4, T6, T12 and T24	Higher volume of distribution and lower half-life compared to other types of ICU patients	IAC = 85% (*n* = 12/14) No unbound concentration, nor peritoneal samples No ATF concentrations during surgery
Garcia-de-Lorenzo [76]	Prospective monocentric PK study Blood and peritoneal samples	Adults with severe burn injuries or complicated IAIs *N* = 10 (IAI) SOFA 5 [1.5–7.5]	Micafungin 1.5 mg/kg (BW)	Day 1, between 3 and 4 h1, h3, h5, h8, h18 and h24 Peritoneal samples obtained from drain	Penetration ratio: 0.29	IAC = 4 No unbound concentration No ATF concentrations during surgery
Grau [70]	Prospective monocentric PK study Blood and peritoneal samples	Adults with PPO *N* = 10 Apache II 15 [12–24] SOFA 5 [1.5–7.5]	Micafungin 100 mg	3 days after MCF initiation Before, h1, h3, h5, h8, h18 and h24 Peritoneal samples issues from a Jackson-Pratt drain	MCF exposure PF < plasma Penetration ratio: 0.3 *C*_*max*_ achieved in 5-8h in the PF 100% PTA: *C. albicans* (MIC 0.016 mg/l) *C. parapsilosis* (MIC 0.25 mg/L)	IAC = 40% (*n* = 4/10) No unbound concentration Peritoneal samples obtained at Day 3 d, from drain No ATF concentrations during surgery
Pea [48]	Case-series Blood, bile, and ascites samples	Transplanted adult patients non-critically ill patients *N* = 3 (1 cholangitis, 2 peritonitis)	Fluconazole LD 400 mg then 100–200 mg q24h depending on renal function	At steady state, from plasma, bile drains or paracentesis for ascitic fluid	Penetration ratio bile: 0.5 Penetration ratio ascites: 0.8	Documented candidiasis 100% Non-critically ill patients No unbound concentration No ATF concentrations during surgery

ANF: anidulafungin; APACHE II score: acute physiology and chronic health evaluation; ATF: antifungal; BW: body weight; C_max_: maximal concentration; CSF: caspofungin; IAI: intra-abdominal infection; IAC: intra-abdominal candidiasis; MCF: micafungin; PF: peritoneal fluid; PK: pharmacokinetic; PPO: post-operative peritonitis; PTA: probability of target attainment; q24: every 24 h; SAPS II score: simplified acute physiology score; SOFA score: sequential organ failure score

##### Which lessons?

Recent data supporting the need for higher echinocandin doses are mostly from PK studies. This data does not demonstrate, however, that the use of these proposed higher echinocandin doses is associated with better clinical outcomes.

Considering the PK variability of echinocandins in the plasma and the low to moderate penetration ratios in the peritoneal fluid, the risk of suboptimal echinocandin exposures at the site of infection remains high. Thus, use of higher doses and TDM are valid considerations. However, there are currently no defined therapeutic ranges for echinocandins [40, 52, 53]. Some authors have proposed a total trough concentration > 1 mg/L or between 1 and 3 mg/L [52, 53].

### Second-line agents

#### Liposomal amphotericin B

The lipid formulations of amphotericin B deoxycholate (L-AmB) are recommended as alternatives in cases of echinocandin-resistant *Candida* infections [10, 11]. L-AmB has many PK/PD advantages such as broad-spectrum coverage, rapid time-kill rate, post-antifungal effect, and action against biofilm formation [77, 78]. PK/PD studies of L-AmB involving critically ill patients are scarce and reported lower Vd with considerable variability in L-AmB concentrations [79–81]. Furthermore, no clear PK/PD target has been defined for the liposomal formulation. Table 1 provides general PK data and influence of RRT [54], ECMO [82–84], and obesity on L-AmB dosing [85]. Considering the high variability of L-AmB concentrations with a high risk of underdosing, it seems reasonable to propose the dose of 5 mg/kg/day in critically ill patients with IAC which has been reported to be safe [86]. Last, one pediatric study has evaluated peritoneal diffusion and reported lower peritoneal L-AmB concentrations than plasma concentrations [87].

#### Voriconazole

Voriconazole is the second most used azole in critically ill patients with IAC [88]. It has been proposed as an alternative option in severe intra-abdominal infections that have a risk of fluconazole-resistant *Candida* strains [12]. Data in critically ill patients are scarce and reported large interindividual variability [89, 90]. PK/PD main characteristics of voriconazole are provided in Table 1 [54, 90] with influence of various clinical situations [13, 91–94] and extracorporeal supports [31, 54, 92, 95–98]. Considering the high interindividual variability and high occurrence of drug-drug interactions, voriconazole TDM is strongly recommended [13, 40]. Last, one study reported a peritoneal penetration ratio of 0.54 and 0.67 for single and multiple doses, respectively (Table 3) [99].

**Table 3:** Overview of pharmacokinetic and pharmacodynamic parameters and dosing regimens of new antifungal agents

	Rezafungin (CD101, Rezzayo™)	Ibrexafungerp (SCY-078, MK-3118)
Class	Echinocandin (structural analog of anidulafungin)	Triterpenoid (Semi-synthetic derivative of enfumafungin)
Indication	Treatment of candidemia and invasive candidiasis, in cases with limited or no alternative antifungal options	Treatment of vulvovaginal candidiasis
Authorities’ approvals	EMA: 2022 FDA: 2023	FDA: 2021
Recommended dosing regimen	LD 400 mg MD 200 mg weekly	LD 1000–1500 mg MD 500–750 mg daily
Mode of administration	intravenous	oral
Bioavailability	Not applicable	35 to 50%
C_max_ (mg/L)	11.8–19.2	0.43
AUC (mg*h/L)	667–827	6.8
Vd (L/kg)	0.95	8.5
Protein binding (%)	87.5–93.6	99.5–99.8
Half-life (h)	152	20–30
CL (mL/min)	5.8	880
*Candida* spectrum	All Including *C. auris* (↓ for *C. parapsilosis*)	All Including *C. auris* (↓ for *C. krusei, lusitaniae* and *guillermondi*)
PK/PD target	*f*AUC/MIC	*f*AUC/MIC
Critically ill patients	No data	No data
Critically ill patients with IAC	No data	No data
Animal model with IAC	Faster, higher, and longer peritoneal diffusion	Excellent penetration in the liver

According to [100–120]

AUC: area under the curve; CL: clearance; C_min_: minimal concentration; C_max_: maximal concentration; *f*AUC: AUC of the free concentration; EMA: European Medicines Agency; FDA: U.S Food and Drug administration; IAC: intra-abdominal candidiasis; IV: intravenous; LD: loading dose; MD: maintain dose; MIC: minimal inhibitory concentration; PK/PD: pharmacokinetic and pharmacodynamic; Vd: volume of distribution

### New antifungals

Among new antifungals arriving through the development pipeline [100], Rezafungin and Ibrexafungerp have been evaluated within phase 3 clinical trials in patients with invasive candidiasis [101–103]. Rezafungin is a new echinocandin with extended half-life and improved tissue penetration compared to other echinocandins [104–106]. The main PK/PD parameters [106, 107] and spectrum of activity [108, 109] are described in Table 2. Rezafungin PK is not affected by age, sex, race, body weight (34–155 kg), renal clearance (9.3 to > 120 ml/min), continuous RRT [110], and impaired liver function (Child Pugh B or C) [111]. The PK/PD properties of rezafungin may advocate its use in IAC. Indeed, as it has been reported that IAC could provide a reservoir for the emergence of *Candida* resistance [112], considering the front-loaded exposure and higher tissue penetration, rezafungin may be associated with a lower risk of emergence of resistance compared to the other echinocandins [113]. However, its half-life precludes dose adjustment before one week. Considering the dynamic process of PK alteration in critically ill patients, clinical studies are warranted to quantify rezafungin exposure at the onset of infection.

Ibrexafungerp inhibits the production of 1.3-beta-glucan through non-competitive inhibition of the 1.3-beta-glucan synthase complex [114, 115]. The main PK/PD parameters [116–119] and spectrum of activity [114, 119] are described in Table 2. No dosage adjustment is recommended in patients with renal and mild-to-moderate hepatic impairment. Excellent tissue penetration has been reported in the liver, lung, kidney, spleen, skin and bone [114]. Regarding IAC, one murine model has confirmed excellent penetration of ibrexafungerp penetration in the liver with prolonged therapeutic exposure [120].

In addition to its interest in echinocandin *Candida* resistant strains, ibrexafungerp could be useful as an oral drug in replacement of azoles for de-escalation, especially when azoles are not well-tolerated. Ibrexafungerp is currently under investigation for step-down therapy after initial empirical treatment with echinocandins (clinicalTrials.gov number NCT02244606).

## Pharmacodynamic considerations

### Mechanisms of antifungal resistance

An increase in fluconazole and echinocandin resistance has been reported in both *C. albicans* and non-albicans species. This increase in antifungal resistance is mainly associated with an increased exposure to antifungal therapy in the ICU [112, 121, 122].

Mechanisms of antifungal resistance depend on the *Candida* species as well as the antifungal [123]. Broadly, there are three main mechanisms of antifungal resistance:

- Presence of biofilm, where the highest MICs have been observed mostly from in vitro studies [124],
- Increased number of efflux pumps which precludes accumulation of antifungal in the fungal cell [125],
- Reduced 1.3 beta-d-glucan synthase sensitivity [125].

These three mechanisms have been observed in *C. albicans.* The reduction in 1.3 beta-d-glucan synthase sensitivity caused by a mutation in FKS1 and FKS2 genes has been demonstrated to confer a cross resistance to azoles and echinocandins. *C. glabrata* has a reduced susceptibility to azoles secondary to an overexpression of efflux pumps [125] and a reduced susceptibility to echinocandins through mutations in the FKS1 or FKS2 genes [126].

### Antifungal resistance in IAC is due to poor tissue diffusion

Studies focusing on IAC are scarce and mostly come from animal model. Zhao et al. [105] reported a poor diffusion within the lesion during the first 6h after a single dose in a murine model of IAC. These results highlighted, especially during the first day of therapy, insufficient drug exposure which potentially promotes development of antifungal resistance. A second study from Cheng et al. [127] used a murine model of IAC to address the virulence of *C. glabrata*. They reported that if the inoculum was not controlled at the onset of infection, it led to a high occurrence of candidemia with 100% of mice death. Then, if *C. glabrata* was not eradicate, abscess formation could occur, which persisted in most of the mice for at least 28 days. This study describes the dynamic process of IAC depending on the size of the inoculum and highlights the importance of adequate antifungal exposure at the onset of IAC to avoid candidemia, and thereafter to avoid abscess and/or tertiary peritonitis where antifungal diffusion remains challenging. One study has evaluated the prevalence of antifungal resistance among patients with IAC and prior echinocandin exposure. FKS mutant *Candida* isolates were identified in 24% (6/25) of patients [128], with the presence of FKS mutations associated with prolonged echinocandin exposure (*P* = 0.01) and therapeutic failures despite source control interventions (100%). The authors suggest that IAC acts as a hidden reservoir for the emergence of echinocandin-resistant *Candida*. These observations were supported by the ongoing challenge of insufficient drug penetration during therapy for IAC supported by animal studies and clinical studies.

### The *C. auris* threat

In the last decade, an increased number of outbreaks involving *C. auris* has been reported worldwide [129, 130]. *C. auris* has reduced susceptibility to the predominantly used antifungals (i.e. azoles, echinocandins, amphotericin B) [131, 132]. Current microbiological data reported that 90% of *C. auris* strains demonstrated resistance to fluconazole, 30% to amphotericin B, and 5% to echinocandins [133]. Rezafungin and ibrexafungerp demonstrated better susceptibility and a reduction in mortality using an animal *C. auris* candidemia model [131]. Thus, considering the risk suboptimal antifungal concentrations during IAC and the ability to develop resistance to antifungal of *C. auris*, the choice of antifungal and dose are of paramount importance in case of IAC caused by this difficult-to-treat pathogen.

### Which role for the immune response?

Lastly, the role of immunological processes during IAC must be considered when determining antifungal PK/PD targets, as both innate and adaptive immunity are important for defence against *Candida* [134]. Neutrophils and macrophages have an important role in *Candida* recognition and activation of the immune response thereafter. As mentioned above, abdominal surgery induced-trauma provides peri- and post-operative inflammation followed by immunosuppression [22]. Impairment of the immune response during this period can promote *Candida* growth and exacerbate virulence [135, 136]. Besides, *Candida* is frequently encountered in post-operative peritonitis [137]. As it has been suggested to aim for higher PK/PD targets for antibiotics in the case of neutropenia [138], we wonder if in post-operative peritonitis, a more aggressive antifungal PK/PD target may need to be considered. Thus, studies including immunosuppressed critically ill patients with IAC exploring the relationship between antifungal exposure and clinical outcomes are also warranted.

**Fig. 1:**
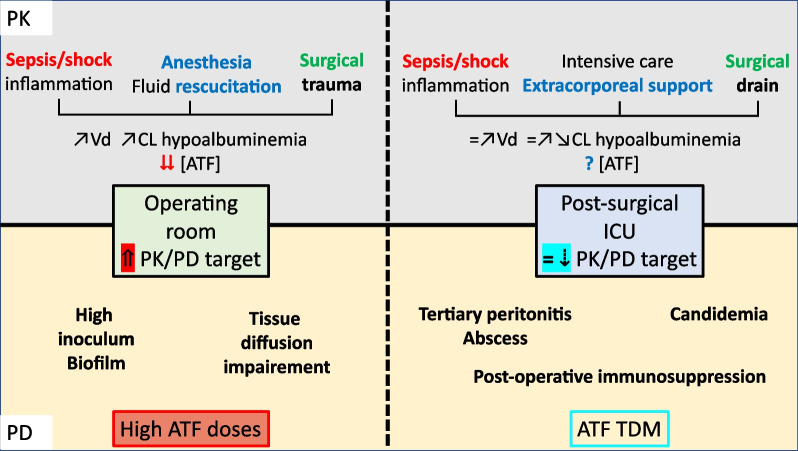
PK/PD alterations during intra-abdominal candidiasis. PK: pharmacokinetic; PD: pharmacodynamic; Vd: volume of distribution; CL: clearance; ICU: intensive care unit; ATF: antifungal; TDM: therapeutic drug monitoring. Figure 1 is split twice: vertically, where left side represents the per-operative phase and right side the post-operative period, and horizontally, where the upper case described the sources of pharmacokinetic alterations, and the lower case, the sources of pharmacodynamic alterations. In the operating room, sources of PK variability are driven by the sepsis/shock, the resuscitation (fluid resuscitation and catecholamines), the anaesthesia, and inflammation caused by the surgical trauma. Together they contribute to a high risk of suboptimal antifungal concentrations by increase in volume of distribution and clearance in both the plasma and the peritoneum. From a pharmacodynamic perspective, before the source control is performed by the surgeon, high inoculum potentially protect by *Candida* biofilm and low tissue penetration could promote antifungal resistance. Thus, increased PK/PD target and high dose of antifungal are required. During the post-operative period, the source control is supposed to be achieved and thereby the sepsis/shock should be less important. However, organ failures caused by the abdominal sepsis could occur, and provide the need for extracorporeal support such as renal replacement therapy or ECMO. The presence of surgical drains could increase drug clearance. Therefore, antifungal concentrations are highly unpredictable, from low to high concentration. From a pharmacodynamic perspective, source control has been performed but an immunoparalysis could be present and candidemia and/or abscess/tertiary peritonitis could occur. Therefore, therapeutic drug monitoring of antifungal should be considered

Figure 1 summarises the PK and PD considerations when treating critically ill patients with IAC requiring surgery.

## Lessons learned from PK/PD studies conducted in critically ill patients with IAC

Table 3 provides a summary of retrieved PK/PD studies which have focused on the use of antifungal during IAC. Details regarding study selection are provided in the Additional file 1 with the ClinPK statement checklist [139] for each included study (Additional file 1: Table S2**)**. Overall, the studies fulfilled more than 80% of PK analysis checklist items (namely, use of valid quantitative bioanalytical methods, description of the PK modelling methods and software used, and adequate detail of the PK analysis performed). The main weakness across all studies was related to patient selection and follow-up from a clinical perspective.

### Population consideration

All study investigators acknowledged the small size of their cohorts (between 3 and 31 patients) and highlighted a high inter-subject variability. Moreover, the case-mix of patients was mostly composed of secondary peritonitis, with IAC suspected rather than confirmed in most cases. Thus, the impact of PK/PD target attainment on mortality cannot be addressed due to the small sample sizes and the low number of confirmed cases of IAC. Echinocandin is the most evaluated antifungal class in IAC. Only one case-series addressed the PK of fluconazole in abdominal samples, in a non-ICU population.

### Laboratory consideration

In all studies, only the total concentration was measured. However, from a tissue diffusion perspective, the unbound concentration should be assessed in further studies, especially in critically ill patients because of a high prevalence of hypoalbuminemia and marked fluctuations in serum albumin concentrations during acute illness [140]. Regarding peritoneal samples, most studies obtained samples from in situ abdominal drains and acknowledged less data compared to blood samples given these drains were usually removed after 72 h post-operatively. Therefore, peritoneal fluid samples obtained intra-operatively would allow for more accurate PK data describing antifungal abdominal diffusion.

### Clinical course of IAC consideration

All studies analysed the PK/PD targets based on antifungal concentrations obtained from the post-operative period, often two to three days after surgery to evaluate steady-state concentrations. In our opinion, except perhaps for tertiary peritonitis (poorly studied thus far), the post-operative period has lower impact on mortality compared to the surgical period. Indeed, mortality increases with delay in source control and commencement of antifungal therapy [4]. Thus, if a PK/PD study aims to evaluate the impact of PK/PD target attainment on mortality, intra-operative sampling must be considered because: (i) this is the phase where inoculum is potentially the highest, (ii) the PK alterations from sepsis, surgery, and resuscitation therapeutics are maximized, and (iii) source control is ongoing.

However, there is still a risk of unpredictable antifungal concentrations during the post-operative period. Indeed, because of all other sources of PK variability in critically ill patients such as renal failure or extracorporeal support, the risk of suboptimal exposure can persist throughout the course of therapy [19, 141]. Therefore, in cases of either candidemia, tertiary peritonitis or inadequate source control, ensuring optimal antifungal exposure is crucial, knowing that these situations require longer antifungal duration [142].

## Further perspectives

Based on the PK and PD challenges noted, we believe that further clinical studies evaluating impact of antifungal PK/PD parameters on outcome such as mortality are warranted and of crucial importance, especially studies that sample during the peri-operative phase during surgical intervention. This observation implies that antifungal therapy should commence before surgery, which is not always the case because of the diagnostic difficulties associated with identifying IAC [143]. Indeed, in the AMARCAND-2 study, antifungal therapy was started after *Candida* documentation in 70% of the patients, thus noting that few patients received antifungal at the onset of IAC [88]. Performing PK/PD analysis during and after surgery would allow to describe important fluctuations in antifungal exposure, some of which may impact on patient outcomes. Certainly, the identification of critically ill patients with intra-abdominal infection who then develop IAC remains a challenge [144, 145]. The future consensus definition of IAC from the FUNDICU project [146] is eagerly awaited and would certainly help identifying the right population.

An additional difficulty when addressing clinical outcome such as mortality in critically ill patients with IAC is related to the *Candida* itself [147–149]. Although the presence of *Candida* in peritoneal samples is associated with poor outcomes [150], other studies have been inconsistent [151]. This may be due to whether the *Candida* isolated is a true pathogen responsible for mortality. Indeed, the pathogenicity of *Candida* has been questioned, with suggestion that it may be dependent on the clinical situation and/or underlying condition of the patient [152–154].

In the interim of obtaining more robust PK/PD data linked to patient outcomes, using antifungal TDM in critically ill patients with IAC, especially in cases with high prevalence of non-*albicans* species, should be advocated. In the absence of clear recommendations specifically for IAC in critically ill patients [52], and based on available PK/PD data [48, 57, 73, 74], we suggest trough concentrations between 10 and 20 mg/L for fluconazole, and 1 to 10 mg/L for echinocandins. Further studies aiming to evaluate these therapeutic ranges in critically ill patients are, however, required.

It is noteworthy that data regarding antifungal dose optimization using dosing software are scarce. In invasive candidiasis, only one study has been reported. Bayesian analysis using a limited sampling strategy has been evaluated for anidulafungin using data from 20 critically ill patients and showed reasonable prediction [69]. Given that use of TDM in combination with dosing software may become more common practice in the ICU, assessment of these dose optimization interventions is a future consideration.

## Conclusion

Due to the specific pathophysiology and associated interventions, IAC must be considered differently compared to other forms of invasive candidiasis such as candidemia. High-quality PK/PD studies are required to better describe the rate of antifungal target attainment in both plasma and peritoneal fluid, during and after the surgery, and when the patient is transferred to the ICU. The lack of intra-operative data is a current weakness. Proposed PK/PD targets, derived mostly from animal models, have yet to be validated in the critically ill population. To study the impact of antifungal PK/PD target attainment on clinical outcomes, larger sample sizes and multicentre studies are needed. In the meantime, antifungal TDM in critically ill patients with IAC should be considered, especially in cases of high prevalence of non-*albicans* species or when fluconazole is prescribed. Regarding the interest in new antifungals, studies involving critically ill patients coupled with rigorous PK/PD analysis are warranted before a more widespread use.

### Supplementary Information

**Additional file 1**. **Supplementary material S1:** Methodology of the literature review for pharmacokinetic studies evaluating antifungals in IAC. **Supplementary material S2:** ClinPK checklist applied to evaluated pharmacokinetic studies.

## Data Availability

Not applicable.

## Abbreviations

AUC: Area under the curve
ECMO: Extra-corporeal membrane oxygenation
*f*AUC: Area under the curve of the free (unbound) concentration
IAC: Intra-abdominal candidiasis
ICU: Intensive care unit
L-AmB: Liposomal amphotericin B
PD: Pharmacodynamic
PK: Pharmacokinetic
RCT: Randomized controlled trial
RRT: Renal replacement therapy
TDM: Therapeutic drug monitoring
Vd: Volume of distribution
