# Supplementary material for: Pharmacokinetic and pharmacodynamic considerations for antifungal therapy optimisation in the treatment of intra-abdominal candidiasis
Source: Crit Care. 2023 Nov 20;27:449. doi: 10.1186/s13054-023-04742-w (PMC10659066; doi:10.1186/s13054-023-04742-w)
Supplement: Supplementary file 1 — Additional file 1. Supplementary material S1: Methodology of the literature review for pharmacokinetic studies evaluating antifungals in IAC. Supplementary material S2: ClinPK checklist applied to evaluated pharmacokinetic studies. [file 13054_2023_4742_MOESM1_ESM.docx]

**Supplementary material S1:** Methodology of the literature review for pharmacokinetic studies evaluating antifungals in IAC:

MEDLINE (via PubMed), EMBASE, and Web of science were searched to identify eligible study to be included for review. The search was performed with no language restriction. The period time analysis was 2020 – 2023 to include studies using recent pharmacokinetic modelling methods. The search time ended in September 2023.

The combination of keywords and search terms used to identify studies were: **Population pharmacokinetic AND echinocandin* OR caspofungin OR micafungin OR anidulafungin OR fluconazol* OR voriconazole OR amphotericin* AND critically ill patients AND intra-abdominal candidiasis OR candida peritonitis.**

**Mesh terms:**

**pharmacokinetic:** "pharmacokinetic"[All Fields] OR "pharmacokinetical"[All Fields] OR "pharmacokinetically"[All Fields] OR "pharmacokinetics"[Subheading] OR "pharmacokinetics"[All Fields] OR "pharmacokinetics"[MeSH Terms]

**caspofungin:** "caspofungin"[MeSH Terms] OR "caspofungin"[All Fields] OR "caspofungine"[All Fields]

**micafungin:** "micafungin"[MeSH Terms] OR "micafungin"[All Fields]

**anidulafungin:** "anidulafungin"[MeSH Terms] OR "anidulafungin"[All Fields] OR "anidulafungin's"[All Fields]

**voriconazole:** "voriconazole"[MeSH Terms] OR "voriconazole"[All Fields] OR "voriconazol"[All Fields] OR "voriconazole's"[All Fields]

**candidiasis:** "candidiasis"[MeSH Terms] OR "candidiasis"[All Fields] OR "candidiases"[All Fields]

**candida:** "candida"[MeSH Terms] OR "candida"[All Fields] OR "candidae"[All Fields] OR "candidas"[All Fields]

**peritonitis:** "peritoneally"[All Fields] OR "peritoneum"[MeSH Terms] OR "peritoneum"[All Fields] OR "peritoneal"[All Fields] OR "peritonism"[All Fields] OR "peritonitis"[MeSH Terms] OR "peritonitis"[All Fields]

Most studies dealing with antifungal PK/PD concerned patients with invasive candidiasis, mostly candidemia. All these studies were carefully checked and only studies reporting data from ICU patients with suspected or confirmed IAC were kept for analysis.

**Type of study to be included:**

Inclusion criteria:

- Pharmacokinetics studies evaluating caspofungin, micafungin, anidulafungin, fluconazole, voriconazole, and liposomal amphotericin B.
- Including all type of intra-abdominal candidiasis and, in case of candidemia, studies will be included only if candidemia was related to intra-abdominal candidiasis.
- Including intensive care population except neonates
- Publication status: only published peer reviewed.

Exclusion criteria:

- Study design: narrative or systematic reviews, and meta-analysis.
- Other invasive candidiasis and candida peritonitis in the context of peritoneal dialysis.
- Publication status: unpublished studies, study protocols, conference papers and abstracts, and studies published without peer-reviews.
- Studies in animals.

The first research led to 624 studies. After reviewing of titles and abstracts, 19 studies were kept for full text review. Among these 19 articles, seven were removed because of the study design (2), evaluating other drug (2) or other ICU population (2). The literature review was performed by EN and MOC and disagreements were resolved through discussion with JAR or CR.

**Supplementary material S2:** ClinPK checklist applied to evaluated pharmacokinetic studies**.**

|  | Pea^[48]^ | Grau^[70]^ | Garcia-Lorenzo^[76]^ | Dupont^[71]^ | Pérez civantos^[75]^ | Gioia^[73]^ | Garbez^[72]^ | Welte^[74]^ | Garbez^[57]^ | Lin^[99]^ | Tortora^[87]^ |
| --- | --- | --- | --- | --- | --- | --- | --- | --- | --- | --- | --- |
| **Title/abstract** |  | | | | | | | | |  |  |
| The title identifies the drug(s) and patient population(s) studied. | **Y** | **Y** | **Y** | **Y** | **Y** | **PY** | **Y** | **Y** | **Y** | **Y** | **Y** |
| The abstract includes the name of the drug(s) studied, route of administration, population in whom it was studied, and results of the primary objective and major clinical pharmacokinetic findings. | **NA** | **Y** | **Y** | **Y** | **PY** | **PY** | **Y** | **PY** | **Y** | **Y** | **Y** |
| **Background** |  | | | | | | | | |  |  |
| Pharmacokinetic data that is known and relevant to the drugs being studied is described | **N** | **N** | **Y** | **N** | **Y** | **N** | **Y** | **N** | **Y** | **Y** | **N** |
| An explanation of the study rationale is provided | **Y** | **Y** | **Y** | **Y** | **Y** | **Y** | **Y** | **Y** | **Y** | **Y** | **Y** |
| Specific objectives or hypotheses is provided | **N** | **Y** | **Y** | **Y** | **Y** | **Y** | **Y** | **Y** | **Y** | **Y** | **Y** |
| **Methods** |  | | | | | | | | |  |  |
| Eligibility criteria of study participants are described | **NA** | **Y** | **Y** | **Y** | **Y** | **Y** | **Y** | **Y** | **Y** | **Y** | **N** |
| Co-administration of study drug(s) with other potentially interacting drugs or food within this study is described. | **N** | **NA** | **NA** | **NA** | **NA** | **NA** | **NA** | **NA** | **NA** | **Y** | **NA** |
| Drug preparation and administration characteristics are described | **N** | **PY** | **Y** | **PY** | **PY** | **PY** | **Y** | **N** | **Y** | **Y** | **N** |
| Body fluid or tissue sampling (timing, frequency and storage) for quantitative drug measurement is described. | **N** | **Y** | **Y** | **Y** | **Y** | **Y** | **Y** | **Y** | **Y** | **Y** | **Y** |
| Validation of quantitative bioanalytical methods used in the study are referenced or described if applicable. | **Y** | **Y** | **Y** | **Y** | **Y** | **Y** | **Y** | **Y** | **Y** | **Y** | **Y** |
| Pharmacokinetic modeling methods and software used are described | **NA** | **Y** | **Y** | **Y** | **Y** | **Y** | **Y** | **Y** | **Y** | **Y** | **NA** |
| For population pharmacokinetic studies, covariates incorporated into pharmacokinetic models are identified and described. | **NA** | **Y** | **Y** | **Y** | **NA** | **NA** | **Y** | **NA** | **Y** | **NA** | **NA** |
| Formulas for calculated variables are provided or referenced. | **N** | **NA** | **Y** | **N** | **N** | **N** | **Y** | **N** | **Y** | **N** | **N** |
| The specific body weight used in drug dosing and pharmacokinetic calculations are reported | **N** | **N** | **Y** | **N** | **N** | **N** | **Y** | **Y** | **Y** | **N** | **N** |
| Statistical methods including software used are described | **NA** | **Y** | **Y** | **Y** | **Y** | **Y** |  | **Y** | **Y** |  |  |
| **Results** |  | | | | | | | | |  |  |
| Study withdrawals or subjects lost to follow-up (or lack thereof) are reported. | **NA** | **N** | **N** | **N** | **N** | **N** | **N** | **N** | **N** | **N** | **N** |
| Quantification of missing or excluded data is provided if applicable | **NA** | **N** | **N** | **N** | **N** | **N** | **N** | **N** | **N** | **N** | **N** |
| All relevant variables that may explain inter- and intra-patient pharmacokinetic variability are provided with appropriate measures of variance. | **No** | **PY** | **Y** | **PY** | **N** | **Y** | **Y** | **N** | **Y** | **Y** | **N** |
| Results of pharmacokinetic analyses are reported with appropriate measures of precision | **NA** | **Y** | **Y** | **Y** | **Y** | **Y** | **Y** | **Y** | **Y** | **Y** | **NA** |
| Studies in patients receiving extracorporeal drug removal (i.e., dialysis) should report the mode of drug removal, type of filters used, duration of therapy and relevant flow rates. | **NA** | **NA** | **NA** | **NA** | **NA** | **N** | **NA** | **N** | **NA** | **NA** | **NA** |
| **Discussion/Conclusion** |  | | | | | | | | |  |  |
| Study limitations describing potential sources of bias and imprecision where relevant should be described | **Y** | **Y** | **Y** | **Y** | **N** | **Y** | **Y** | **Y** | **Y** | **Y** | **Y** |
| The relevance of study findings is described | **Y** | **Y** | **Y** | **Y** | **Y** | **Y** | **Y** | **Y** | **Y** | **Y** | **Y** |
| **Other Information** |  |  |  |  |  |  |  |  |  |  |  |
| Funding sources and conflicts of interest for the authors are disclosed. | **Y** | **Y** | **Y** | **Y** | **Y** | **Y** | **Y** | **Y** | **Y** | **Y** | **Y** |

Abbreviations: Y: Yes; PY: Partially Yes; N: No; NA: not applicable.

According to Kanji S, *et al*. [139] “Reporting Guidelines for Clinical Pharmacokinetic Studies: The ClinPK statement”.
